# Disease-specific quality of life as part of the long-term follow-up for children born with esophageal atresia in an academic unit in South Africa—a pilot study

**DOI:** 10.1093/dote/doae016

**Published:** 2024-03-12

**Authors:** C de Vos, M Dellenmark-Blom, F M Sikwete, D Sidler, L van Wyk, P Goussard

**Affiliations:** Division of Pediatric Surgery, Stellenbosch University and Tygerberg Hospital, Cape Town, South Africa; Department of Pediatrics and Child Health, Stellenbosch University and Tygerberg Hospital, Cape Town, South Africa; Department of Pediatric Surgery, The Queen Silvia Children’s Hospital, Gothenberg, Sweden; Division of Pediatric Surgery, Stellenbosch University and Tygerberg Hospital, Cape Town, South Africa; Division of Medical Ethics and Law, Stellenbosch University, Cape Town, South Africa; Department of Pediatrics and Child Health, Stellenbosch University and Tygerberg Hospital, Cape Town, South Africa; Department of Pediatrics and Child Health, Stellenbosch University and Tygerberg Hospital, Cape Town, South Africa

**Keywords:** esophageal atresia, long-term follow-up, quality of life

## Abstract

As neonatal mortality rates have decreased in esophageal atresia (EA), there is a growing focus on quality of life (QoL) in these children. No study from Africa has reported on this topic. This pilot study aimed to describe disease-specific QoL in EA children and its applicability as part of long-term follow-up in an academic facility in South Africa. Disease-specific QoL in children born with EA was assessed utilizing the EA-QoL questionnaire for children aged 2–17 years during a patient-encounter. The parent-report for children aged 2–7 years compromised 17 items categorized into three domains: eating, physical health and treatment, and social isolation/stress. The 24-item EA-QL questionnaire for children aged 8–18 (child- and parent-report) explored four domains: eating, body perception, social relationships, and health and well-being. A total of 13 questionnaires for children aged 2–7 years were completed by five parents. A negative perceived impact on their child’s eating was reported by 46–92% of parents, and less impact in the other two domains. A total of 27 questionnaires were completed by eight children aged 8–17 years and 10 parents. Similar percentages children and parents reported a negative impact in the eating, social relationships, and body perception domains. More than half reported a negative impact on the child’s health and well-being. This study supports the concept that assessment of disease-specific QoL should play a vital role in the comprehensive follow-up approach for children born with EA. We identified that parents of younger children were more likely to report eating disorders, whereas parents of older children were more likely to report health difficulties with different perceptions when it came to the child’s scar.

## BACKGROUND

The concept of health, as defined by the World Health Organization (WHO), encompasses not only the absence of disease and infirmity but also complete physical, mental, and social well-being.^1^ As neonatal mortality rates after surgical repair of esophageal atresia (EA) have decreased, there is a growing focus on the long-term outcomes of children with a history of this condition.^2^ These long-term outcomes include gastrointestinal (GI) symptoms such as esophageal dysmotility, gastro-esophageal reflux, feeding difficulties (e.g. prolonged eating time, vomiting, or choking) and respiratory disorders such as recurrent airway infections and coughing as well as reduced exercise capacity.^2^ All these factors may impact the quality of life (QoL), particularly in school-aged children, which is becoming more important, especially since QoL has been recognized as an essential outcome by expert stakeholders such as the International Network of Esophageal Atresia.^2–4^

QoL refers to an individual’s perception of their overall life situation, considering their expectations and the factors that may limit their well-being.^2^ An age-adapted, disease-specific QoL questionnaire has been developed and tested in Sweden and Germany for children born with EA.^5^ This EA-QoL questionnaire has been shown to be a valid and reliable measure for assessing the health outcomes specific to children born with EA. The EA-QoL questionnaires have since shown acceptable validity in Turkey, Poland, and China and are currently being tested in different countries and languages as part of a large cross-cultural study.^6–10^

Our pilot study aimed to determine the QoL of children born with EA in an academic health care unit in South Africa and to evaluate its suitability as a tool for long-term follow-up of children born with EA. Should it be a suitable tool, a more extensive multicenter study across South Africa will be planned as a future directive.

## METHODS

Ethical approval (HREC: S20/10/260) was obtained from the Health Research Ethics Committee before the onset of the study. A cross-sectional descriptive QoL study of children born with EA, aged 2–17 years, was conducted during follow-up visits at an academic health care unit, between 2021 and 2023. The study evaluated the suitability of using the items included in the EA-QoL questionnaires as a source for narrative information on the child’s functioning and well-being.

### Setting and participants

Standardized follow-up of EA patients in our center occurs annually unless otherwise specified, with no multidisciplinary team approach. For this study we invited all children (depending on their age) born with EA, seen during either routine followed-up or admission to our unit, as well as their parents to participate in an interview-based assessment utilizing the EA-QoL questionnaire. To be included in the study, the children of parents participating needed to be at least 2 years old at follow-up. Children completing the questionnaires themselves needed to be at least 8 years old. All participants were required to provide informed consent and/or assent. Additionally, participants were allowed to complete the EA-QoL questionnaire multiple times, providing that their visits were more than 4 weeks apart.

### Clinical and demographic data

Demographic data, details of associated anomalies, and details of the original surgery and additional surgeries were collected and documented from the children’s hospital records.

### The EA-QoL questionnaires

The EA-QoL questionnaires, which have undergone a rigorous development process in accordance with established principles, were used to evaluate the age-appropriate QoL of our children born with EA.^5^^,^^11^

The EA-QoL consists of three distinct questionnaires, tailored to different age groups: the parent-report for parents of children 2–7 years, the child-report for children 8–17 years old and the parent-report for children 8–17 years old.

The parent-report questionnaire for children aged 2–7 years comprises 17 items, categorized into three domains: eating, physical health and treatment, and social isolation. Additional to each domain score, the scores of the domains can be combined to generate a total score, providing an overall assessment of the child’s QoL within this age range. The child-and-parent-report for children 8–17 years old comprises 24 items, categorized into four domains (eating, body perception, social relationships and health, and well-being) and can be summarized to a total score. The included individual items can also be used to provide descriptive information on the child’s QoL. All questions were answered on a 5-point Likert scale: never, rarely, sometimes, often, or always and were answered using a recall period of 4 weeks.

Following forward–backward translation according to the well-established principles, these questionnaires went through different phases of development to formulate a questionnaire that could be tested and used in our center.^12^

### Data collection and statistical considerations

Descriptive statistics were employed for data analysis. Categorical variables were analyzed using frequency and percentages, while numerical variables were assessed by calculating means and standard deviations. To further quantify the reported impact in the children’s lives in the previous 4 weeks, replies on the EA-QoL questionnaires were categorized into negatively perceived impact (rarely, sometimes, often, always) and no perceived impact (never), in addition to the item distribution on the 5-point Likert scale. The narrative content of the respondents’ answers on the 5-point Likert scale was presented and described for each domain.

## RESULTS

The study enrolled a total of 23 participants including 15 parents and 8 children, who completed a total of 40 EA-QoL questionnaires during the study period. Five mothers of five children (three males and two females) completed the parent-report questionnaire for children 2–7 years a total of 13 times. The mean ages of the children and mothers at the time of completion of the questionnaires in this group were 40 ± 15 months and 35 ± 15 years, respectively.

A total of 10 parents (eight mothers and two fathers) completed 14 parent-reports for children 8–17 years, all of whom reported being in good health without any personal health complaints, except for one mother. Eight children (five males and three females), aged 8–17 years, completed the child-report questionnaire, resulting in 13 completed questionnaires. The parent’s median age at the time of completion was 43 ± 9 years, and the children’s age was 11 ± 2 years.

**Fig 1:**
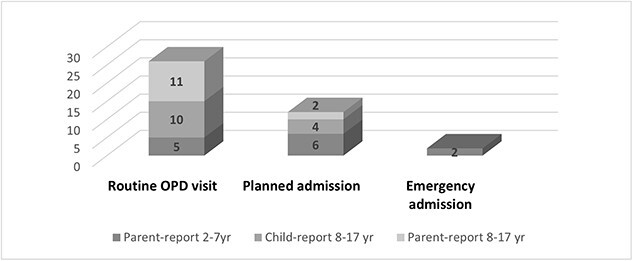
Reason for visit during which the EA-QoL questionnaire was completed

Details of the demographic and clinical data for children born with EA are provided in Table 1. Their mean weight at time of follow-up was 14 ± 11 kg, and their mean height was 110 ± 37 cm. The reason for their visits is documented in Figure 1.

**Table 1:** Demographic and clinical data of EA children enrolled

**Birth history**	**Results**
• Prenatal diagnosis made; n (%) • Birth weight; mean ± SD* • Gender; % (male: female) • Gestational age; mean ± SD • One of twins; n (%)	• 1 (7) • 2477 g ± 657 g • 57:43 • 36 ± 3 weeks • 2 (14)
Type of anomaly	
• EA only; n (%) • EA with a distal TEF; n (%) • Associated anomaly present; n (%)	• 2 (14) • 12 (86) • 9 (64)
Details about the surgery	
• Age of repair; mean ± SD • Primary repair done; n (%) • Elongation or esophageal replacement surgery done; n (%) • Revision surgery for recurrent TEF or anastomotic leak; n (%) • Patients who had a gastrostomy at any stage; n (%) • Sepsis as complication; n (%)	• 14.9 ± 20.1 days • 13 (93) • 5 (36) • 4 (29) • 6 (43) • 2 (14)
Number of dilatations to date; mean ± SD	• 3 ± 2

### Parent-report (2–7 years)

#### Negatively perceived impact on the child’s QoL

**Fig 2:**
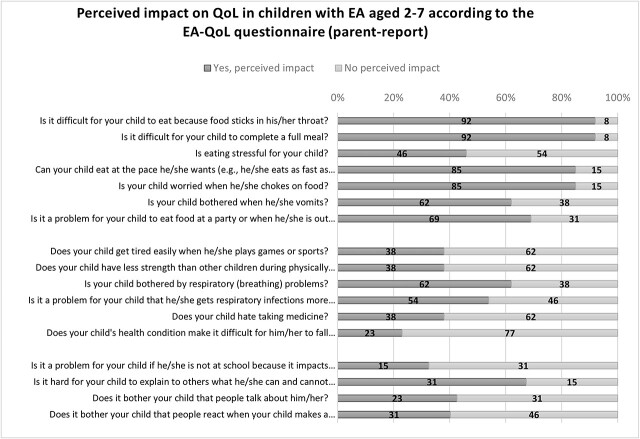
Negatively perceived impact on QoL (parent-report 2–7 years)

Figure 2 presents the negatively perceived impact on QoL in children with EA aged 2–7 according to the EA-QoL questionnaire (parent-report). Parents reported a wide range (46–92%) of a negatively perceived impact on their child’s eating, 23–62% reported a negatively perceived impact on their child’s physical health and treatment, and 15–31% on their child’s social lives in the 4 weeks prior to answering the questionnaire.

#### Narrative descriptive information

The most common responses from mothers were that eating was *never* stressful for their child (54%), that their child was *rarely* bothered by vomiting (46%) or worried when he/she chokes on food (38%), that it was *sometimes* difficult for their child to eat because food sticks in his/her throat (46%), to complete a full meal (46%), to eat at the pace he/she wanted (31%), and *sometimes* problematic to eat food at a party or when he/she is out with friends (31%). Table 2 details the rest of the results of the EA-QoL questionnaire for children aged 2–7 years according to the 5-point Likert scale never to always (parent-report).

**Table 2:** Results of the parent-report for children 2–7 years old

**Questions**	**% (*n* = 13)**					
	**5-point response scale**					
	**Never**	**Rarely**	**Sometimes**	**Often**	**Always**	**Too young**
Eating						
Is it difficult for your child to eat because food sticks in his/her throat?	8	38	46	0	8	
Is it difficult for your child to complete a full meal?	8	31	46	0	15	
Is eating stressful for your child?	54	31	8	8	0	
Can your child eat at the pace he/she wants (e.g. he/she eats as fast as he/she wants)?	15	15	31	31	8	
Is your child worried when he/she chokes on food?	15	38	31	15	0	
Is your child bothered when he/she vomits?	38	46	8	8	0	
Is it a problem for your child to eat food at a party or when he/she is out with friends?	31	15	31	15	8	
Physical health and treatment						
Does your child get tired easily when he/she plays games or sports?	62	8	15	0	15	
Does your child have less strength than other children during physically demanding activities (not as strong as others)?	62	15	15	8	0	
Is your child bothered by respiratory (breathing) problems?	38	31	23	8	0	
Is it a problem for your child that he/she gets respiratory infections more often than others?	46	31	15	8	0	
Does your child hate take medicine?	62	15	0	8	15	
Does your child’s health condition make it difficult for him/her to fall asleep or stay asleep at night?	77	8	8	8	0	
Social isolation and stress						
Is it a problem for your child if he/she is not at school because it impacts his/her life negatively?	31	8	8	0	0	54
Is it hard for your child to explain to others what he/she can and cannot do?	15	8	23	0	8	46
Does it bother your child that people talk about him/her?	31	8	0	15	0	46
Does it bother your child that people react when your child makes a noise?	46	23	8	0	0	23

### Parent and child-report (8–17 years)

#### Negatively perceived impact on the child’s QoL

**Fig 3:**
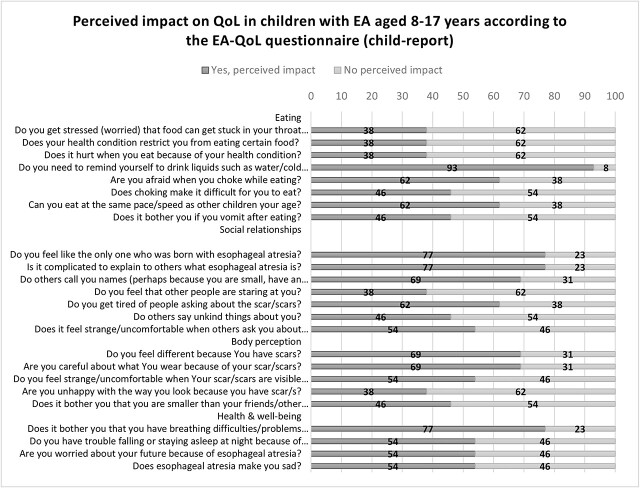
Negatively perceived impact on QoL of children aged 8–17 years (child-report)

**Fig 4:**
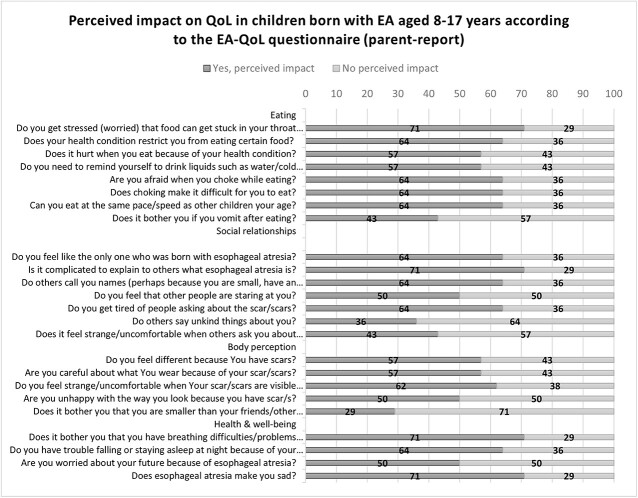
Negatively perceived impact on QoL of children aged 8–17 years (parent-report)

Figures 3 and 4 describe the negatively perceived impact on QoL of children aged 8–17 years. As seen, 38–93% of the children and 43–71% of parents reported a negatively perceived impact on the children’s eating, 38–77% of children and 36–71% of parents on the children’s social relationships, and 38–69% of the children and 29–62% of parents on the children’s body perception. More than half (54–77% of the children and 50–71% of parents) reported a negatively perceived impact on the children’s health and well-being.

#### Narrative descriptive information

Almost half of the children (46%) reported that they *always* felt different due to their scars, 54% were *always* cautious about their clothing choices, and 38% *often to always* felt uncomfortable when their scar/scars were visible to others. However, some parents (43%) stated that their children *never* felt different or paid much attention to what they wore because of their scars and 38% that their children *never* felt uncomfortable when their scars were visible to other people. In terms of being unhappy with their appearance because of their scars, 50% of the parents and 62% of the children agreed that their children themselves did not experience such unhappiness. Table 3 gives an overview of the descriptive results of the EA-QoL questionnaire for children aged 8–17 (child and parent-report).

**Table 3:** Results of the child and parent-report for children aged 8–17 years

**Questions**	**Child-report % (*n* = 13)**					**Parent-report % (*n* = 14)**				
	**5-point response scale**					**5-point response scale**				
	**Never**	**Rarely**	**Sometimes**	**Often**	**Always**	**Never**	**Rarely**	**Sometimes**	**Often**	**Always**
Eating										
Do you get stressed (worried) that food can get stuck in your throat when you eat?	62	0	23	8	8	29	14	29	7	21
Does your health condition restrict you from eating certain food?	62	15	8	0	15	36	0	50	0	14
Does it hurt when you eat because of your health condition?	62	8	15	0	15	43	7	29	14	7
Do you need to remind yourself to drink liquids such as water/cold drink when you eat?	8	8	31	0	54	43	14	7	21	14
Are you afraid when you choke while eating?	38	0	23	15	23	36	14	21	7	21
Does choking make it difficult for you to eat?	54	15	8	8	15	36	36	14	0	14
Can you eat at the same pace/speed as other children your age?	38	0	38	0	23	36	29	14	0	21
Does it bother you if you vomit after eating?	54	15	15	0	15	57	21	7	7	7
Social relationships										
Do you feel like the only one who was born with EA?	23	8	15	0	54	36	21	14	7	21
Is it complicated to explain to others what esophageal atresia is?	23	0	0	31	46	29	14	21	7	29
Do others call you names?	31	8	15	15	31	36	21	14	21	7
Do you feel that other people are staring at you?	62	0	23	8	8	50	7	14	14	14
Do you get tired of people asking about the scar/scars?	38	15	23	8	15	36	0	29	21	14
Do others say unkind things about you?	54	15	8	8	15	64	7	7	7	14
Does it feel strange/uncomfortable when others ask you about esophageal atresia?	46	8	8	15	23	57	7	7	7	21
Body perception										
Do you feel different because You have scars?	31	8	0	15	46	43	0	21	14	21
Are you careful about what You wear because of your scar/scars?	31	0	0	15	54	43	14	14	0	29
Do you feel strange/uncomfortable when Your scar/scars are visible to others?	46	8	8	23	15	38	23	8	8	23
Are you unhappy with the way you look because you have scar/s?	62	8	8	8	15	50	0	21	14	14
Does it bother you that you are smaller than your friends/other children your age?	54	15	15	8	8	71	7	7	7	7
Health & well-being										
Does it bother you that you have breathing difficulties/problems when you exercise?	23	15	38	8	15	29	21	14	29	7
Do you have trouble falling or staying asleep at night?	46	0	54	0	0	36	14	21	21	7
Are you worried about your future because of EA?	46	23	8	8	15	50	7	7	21	14
Does EA make you sad?	46	15	8	23	8	29	14	43	7	7

## DISCUSSION

This study aimed to investigate the disease-specific QoL as part of a long-term follow-up model for children born with EA in an academic unit in South Africa. The study found that the EA-QoL questionnaires generally applied to our patient population, identifying perceived problems that children with EA may encounter daily. Most research on QoL in children with EA has been conducted in Europe, and to the best of our knowledge, this is the first study of its kind from Africa.^13^^,^^14^

As such, the importance of our study should be viewed from the perspective of the United Nations Convention on the Rights of the Child (UNCRC).^15^ Already in 1989, it emphasized that all children have human rights and the right to participate in decisions regarding their own healthcare.^15^ Healthcare professionals have acknowledged the challenges of working with children, particularly those with rare or chronic health conditions such as EA.^16^ Considering the unique challenges posed by rare diseases, a group in Western Australia explored the applicability of the UNCRC, specifically to children born with rare diseases.^17^ Their findings concluded that the UNCRC can be applied to this group of children, highlighting that rare diseases affect their health and fundamental human rights.^17^ EA is an example of such a rare disease, affecting between 1 in 2500–3000 live births globally.^18^ While neonatal complications of EA are well known, there is a growing focus on the long-term morbidity associated with this disease, specifically impacting the QoL of both the children and their families.^19^

A study published by Ure *et al*.^20^ from Germany in 1998 examined the long-term QoL in adult patients born with EA. The study included patients with a mean age of 25 years utilizing a standardized, self-reported questionnaire consisting of 41 items focusing on the GI and pulmonary systems. They used two separate scoring systems (the Spitzer score and GI QoL Index) and found lower scores for both of these in patients with long gap EA compared with those who had a primary anastomosis, as well as healthy volunteers. While the study indicated that the type of EA may impact overall QoL, it also suggested that other variables could contribute to this finding, emphasizing the need for further research and a disease-specific questionnaire for patients born with EA.^20^

It took many years after this article was published in 1998, until such a questionnaire was developed specifically for children born with EA. In 2016, Dellenmark-Blom *et al*.^11^ aimed to present a framework and developed a condition-specific health-related QoL questionnaire for children born with EA. Two years later the same authors described the feasibility, validity, and reliability of an EA-specific QoL questionnaire for children born with EA in Sweden and Germany.^5^ Their study included 124 families, and the EA-QoL questionnaires demonstrated satisfactory psychometric performance specific to this disease.^5^ The authors believed that the use of these questionnaires could improve the overall clinical outcome of children born with EA. Subsequent studies in Turkey, Poland, and China confirmed the applicability of the EA-QoL questionnaire in assessing condition-specific QoL in children born with EA.^6^^,^^8^^,^^9^ The International EA-QoL group presented its initial results at the Quad conference in 2022, further supporting the usefulness of this assessment tool.^10^^,^^21^

Children born with EA are more likely to develop feeding difficulties when compared with their healthy counterparts. Long mealtimes, refusal to eat, and coughing, choking, or vomiting during or after feeds are only some of the challenges faced by these children.^22^ In our study the parents of children aged 2–7 years mostly reported a negative impact on eating. In comparison, more children than parents in the 8–17 years age group reported eating being negatively affected. In 2022, Ardenghi *et al*.^23^ conducted a QoL study of children born with EA in their unit. Similar to our results, they reported worse scores in the “eating’ category in younger children in their cohort.^23^ We would concur with them in concluding that older children develop skills in coping with eating e.g. reminding themselves to drink fluids when eating (more than half of children always reminded themselves to drink fluid with meals) and to eat slower.^23^ In addition, 38% and 54%, respectively, of the older children in our cohort were never bothered by choking or vomiting while eating, which might indicate their adaptation to these feeding difficulties as they get older.

In comparison with other EA-QoL studies, parents in our study reported less impact on physical health in the younger age group.^5^^,^^6^ These other studies have found this to be one of the most affected domains (especially respiratory symptoms) in children aged 2–7 years. The mean age of children in this group in our cohort was 40 months, which might mean that parents are still adjusting to the disease and are not yet familiar with all the disease-related symptoms. When compared with the older age group, this statement might have more value as most parents and children reported a negative impact on health and treatment in this group.

Social interactions only seemed to have a significant negative impact (reported by both children and parents) in the older age group in our cohort, with less than half of parents reporting that their children (aged 2–7 years) were still too young to answer this section of the questionnaire. This is different from the study conducted by Ardenghi *et al*.^23^ who reported lower QoL scores for the younger age group in this domain also finding a statistically significant difference between the long gap and non-long gap groups in their cohort. We were not able to determine this in our research. Final reports for the EA-QoL working group might assist with this in the future.^10^

The EA-QoL questionnaires were developed based on patient and parent input and adapted for the child’s age, resulting in the inclusion of a body perception domain for children aged 8–17 years.^5^^,^^11^ The literature reports that body concerns in children and adolescents with EA are common when judged by the patients themselves. These findings are in line with several studies conducted in adults born with EA, which showed a negatively perceived impact when it came to body perception.^24–27^ The incidence of body dysmorphic disorders (BDDs) in all children globally is on the increase with long-term mental health issues including depression and even suicide.^28^ Surgical scars are one of the conditions that can lead to BDD, and these can either separately or in combination have a negative impact on the mental health of patients.^29^ A study conducted in 2023 reported on the daily QoL impact of scars on patients.^29^ Patients in their cohort had to be at least 18 years old with at least one traumatic or surgical scar. Sixty-eight percent of patients interviewed had major post-surgical scars and although their cohort only included adults and did not specifically focus on different types of surgery, eight themes emerged, including psychosocial well-being of patients.^29^ Some of the subthemes are applicable to the results presented in our study and include impact of the scars on self-image, talking about the scar with other people and their reaction to the scars as well as clothing limitations due to the scars. Forty-six percent of children (8–17 years old) in our cohort reported always feeling different because of their scars and 54% were always careful about what they wore because of it. An interesting observation was the fact that 43% of parents in both these questions felt that this never bothered their children which might highlight the subconscious effect scars might have on the children themselves. In previous Swedish–German studies, agreement between child and parent ratings in the children’s health-related QoL has been good, especially for condition-specific QoL; however least agreement was found in the body perception domain, highlighting the need for bigger multicenter studies.^30^^,^^31^ Thoracoscopic approaches have been proven to have superior cosmetic results compared with the open EA repair, currently still used in our unit.^32^ With the high negatively perceived impact reported by our EA patients in this domain, we should explore changing our surgical approach in the future.

### Health status of children born with EA

One other concept worth mentioning and recognized by the WHO is the Health Status (HS) of children born with EA. HS focuses on the potential limitations and well-being of the child, specifically their physical, mental, and social functioning.^2^ In 2021 Kate *et al*.^2^ conducted a study comparing the HS and QoL of school-aged children born with EA. They showed that although the HS improved in children born with EA between 8 and 12 years old, the QoL decreased in this age group. They did acknowledge that HS and QoL should be considered as two different, but important concepts, in children born with EA, and is an aspect worth exploring.

### Limitations and future direction

The study has several limitations that should be acknowledged. Firstly, the sample size was small, and the study was conducted within a single unit. The main objective of this study was not to provide a comprehensive description of the various developmental stages of a unit-specific version of the EA-QoL questionnaire. Certain modifications of the EA-QoL, primarily of a grammatical nature, may have had an impact on the respondent’s answers. These particular results are all documented as part of a larger cross-cultural multicenter study that is still being finalized. Additionally, we included multiple descriptions of QoL of the same child over time. As the act of completing a QoL questionnaire could be seen as an intervention itself, potentially leading to subsequent interventions aimed at improving the child’s QoL, it is possible that these factors could have influenced the study findings, potentially biasing the results toward better QoL outcomes. For this pilot study we did not specifically look at the impact of multiple visits on the QoL of these patients but is something that should be explored in the future. Our patients had different surgical approaches depending on their type of EA and co-morbidities as seen in Table 1. Due to the small numbers of our pilot study, we could not identify the specific impact of different types of EA or surgical approaches on the impact of our cohort, and important factor needing inclusion in the follow-up, multicenter study. It is worth mentioning that we did not utilize a validated summative scoring model with scale scores from 0 to 100. Instead, we employed a descriptive approach by analyzing individual items using a 5-point Likert scale.^5^^,^^33^^,^^34^

There is a continued need for larger scale studies. To address limitations encountered in previous studies, ongoing international multi-center studies are being conducted to investigate the QoL in children with EA.^10^ These studies should not only examine the QoL of children born with EA but also investigate different subtypes of EA. Additionally, further research should explore the caregiver’s perspective on the HS of this group of children.

## CONCLUSIONS

This study supports the concept that assessment of disease-specific QoL should play a vital role in the comprehensive follow-up approach for children born with EA in South Africa. Using the EA-QoL questionnaire in a patient encounter, we identified that parents of younger children were more likely to report eating disorders, whereas parents of older children were more likely to report health difficulties. In older children, parents and children reported different perceptions of the child’s scar.

A comprehensive understanding of the disease, its potential complications, and its long-term impact on children’s QoL can help parents and caregivers address these issues proactively. This, in turn, may significantly enhance the emotional well-being of the children, their parents, and the entire family. Specifically, seeking disease-specific support groups and engaging in counseling sessions with members of the treatment team may aid in understanding their children’s differing perspectives on the disease.

Considering the significant findings of the EA-specific QoL in our pilot study, its inclusion in the long-term follow-up program for children born with EA is recommended. Parents and/or children should complete the questionnaire at least once a year. Additionally, when appropriate, children and parents should be referred to other team members for psychological evaluation and targeted support. To obtain more robust evidence, larger multicenter studies on the implementation of condition-specific QoL questionnaires in clinical practice of patients with EA are encouraged. Multidisciplinary teams are needed to address all the different aspects of EA patients and should include regular assessment of their QoL, even in resource-restricted environments.

## DECLARATIONS

### Availability of data and material

The datasets used and/or analysed during the current study are available from the corresponding author on reasonable request.

## Notes

Financial disclosure: The authors declare that no funds, grants, or other support were received during the preparation of this manuscript. The authors have no relevant financial or non-financial interest to disclose.
